# The relationship between task value of learning English and English achievement among art majors: A moderated chain mediation model analysis

**DOI:** 10.1371/journal.pone.0349225

**Published:** 2026-05-15

**Authors:** Yan Qin, Xiaoming Wang, Bocun Zhang, Yue Zhang, Xiaoke Xie, Shihua Mao, Juan Wang, Yupeng Yang

**Affiliations:** 1 Department of College English Teaching, Qufu Normal University, Qufu, Shandong, China; 2 College of Chinese Language and Literature, Qufu Normal University, Qufu, Shandong, China; 3 School of Psychology, Qufu Normal University, Qufu, Shandong, China; 4 School of Physics and Physical Engineering, Qufu Normal University Qufu, Qufu, Shandong, China; Ladoke Akintola University of Technology Teaching Hospital: LAUTECH Teaching Hospital, NIGERIA

## Abstract

Art majors often face a distinct learning context for English as a Foreign Language (EFL) learning, characterized by structural and cognitive barriers that increase the difficulty of engaging in English study. However, the motivational mechanisms linking task value of learning English to English achievement within this specific context remain underexplored. This study employed the expectancy-value-cost theory (EVCT) to investigate the role of task value in their unique learning context. By testing a moderated chain mediation model, it systematically analyzed the relationships among task value of learning English, learning engagement, learning procrastination, self-efficacy, and English achievement. An online survey was administered to 588 art majors from a university in China. Data were analyzed using SPSS 26.0, AMOS 24.0, and the PROCESS macro (Model 6 and 91) with bootstrap methods. The results revealed that task value was associated with English achievement not directly, but through a sequential pathway: it was positively related to learning engagement, which in turn was linked to reduced procrastination, and ultimately to better achievement. Learning engagement emerged as a pivotal sequential mediator, whereas procrastination did not mediate the relationship independently. Furthermore, self-efficacy moderated the association between learning engagement and procrastination, with a stronger negative link observed among students with high self-efficacy. These findings highlight the importance of fostering engagement and building self-efficacy to translate task value into successful learning outcomes for art majors, offering practical implications for instructional design in this unique context.

## Introduction

The deepening of globalization has reinforced the significant role of English in tertiary education, linking proficiency to both academic exchange and future career competitiveness [1,2]. Consequently, understanding the drivers of English learning outcomes has generated substantial research, particularly within university-level EFL contexts [3–5]. A prominent theoretical lens for understanding these drivers is Wigfield and Eccles’s Expectancy-Value Theory (EVT) [6]. Building on this foundation, a refined extension, the Expectancy-Value-Cost Theory (EVCT), has been developed, which delineates subjective task value as comprising three positive appraisal dimensions (attainment, intrinsic, and utility value) while explicitly conceptualizing cost as a separate, parallel construct [7,8]. In line with this framework, the present study defined task value as this unidimensional positive appraisal construct. Research grounded in this theoretical tradition has effectively linked the positive appraisal of task value [9–11], along with related constructs such as self-efficacy [12,13], learning engagement [2,14,15], and procrastination [15–17], to language learning outcomes.

Despite the wealth of research within this theoretical tradition, studies have predominantly focused on non-arts majors [2,9,16,17]. In contrast, the EFL learning experiences of art majors, a population noted for distinct pedagogical structures and learning preferences [18,19], remain relatively underexplored. This lack of research raises a critical question regarding the external validity of existing models: To what extent are the motivational and behavioral pathways outlined in this framework applicable to art majors? The question is especially salient given that the English learning context for art majors is marked by unique structural and cognitive challenges that may significantly increase the difficulty of engaging in EFL learning [18,20,21]. Crucially, key constructs within this framework, such as task value, self-efficacy, and their associated learning behaviors, are understood to be context-sensitive [3,10,22–25].

The distinct learning context of art education, combined with the context-sensitive nature of these motivational constructs, constitutes a critical setting for examining the mechanisms and boundary conditions of the framework. Understanding how task value translates into English achievement through sequential behavioral pathways and how this process is moderated by self-efficacy is therefore essential for both theoretical advancement and pedagogical practice in this unique learning environment. Accordingly, this study investigated these dynamics by proposing and testing an integrated moderated chain mediation model that systematically examined the sequential mediating roles of learning engagement and procrastination, as well as the moderating role of self-efficacy.

The innovative value of this study is twofold. By focusing on art majors, a population navigating unique structural and cognitive challenges in EFL learning, this study extends the application of EVCT to an underexplored context, thereby addressing concerns regarding the external validity of existing motivational models that have been predominantly developed and validated with non-arts majors. Additionally, by advancing an integrated model that simultaneously examined the sequential mediation pathway through which task value translates into English achievement and the boundary condition under which this pathway operates, this study moved beyond the isolated or pairwise examinations that have characterized prior research. These innovations not only address the aforementioned research gaps but also provide practical insights for EFL instruction tailored to art students.

## Literature review and hypotheses development

### Art majors

Art students are defined as a distinct group of learners characterized by their chosen academic specializations [26]. Their challenges in EFL learning are rooted in an interconnected system of structural, motivational, and cognitive factors that set them apart from non-art majors.

From a structural and motivational perspective, China’s college admission mechanism for art majors has long prioritized specialized performance, with academic scores serving primarily as a threshold [20]. This institutional focus, combined with intensive specialized training schedules at the university level [20], frames English learning within a context of high competition for time and effort.

This structure means that time devoted to English is often viewed as time taken away from essential professional practice, thereby increasing the difficulty of English learning. It implicitly signals that professional skills hold greater priority than English proficiency [20,21].

At a deeper cognitive level, a significant misalignment exists between art students’ typical learning preferences and the demands of traditional EFL instruction. Art majors often exhibit cognitive styles oriented toward visual processing, hands-on practice, and immediate feedback [18,27,28]. In contrast, conventional English language teaching frequently emphasizes verbal logic, textual analysis, and abstract reasoning [28]. According to Cognitive Load Theory [29], this misalignment generates high extraneous cognitive load, forcing students to expend substantial mental resources to adapt to the instructional format itself, rather than on absorbing and internalizing the language content. This sustained and demanding cognitive adaptation process not only depletes mental energy but also readily erodes the intrinsic value of the learning activity and increases frustration, making them more prone to disengage from tasks [18,20,21].

A potent motivational counterforce, however, arises from the task value, particularly its utility component. The perceived utility of English is a pivotal element of art students’ task value and plays a key motivating role for this population [20,21,30,31]. In an era of globalization, art students are increasingly aware that English proficiency serves as a critical passport for professional development, such as accessing international literature, participating in overseas exhibitions, or collaborating in globalized art markets [30,31]. This recognition is even reflected in the higher education curriculum, as evidenced by the widespread establishment of specialized courses such as “English for Art Majors” in many Chinese universities [20], highlighting the institutional acknowledgment of its importance. This is also corroborated by findings that art students’ perceived value of English and their level of participation are key factors affecting achievement [20,21]. Thus, this strong, externally anchored perception of utility provides a potent motivational resource to partially overcome the aforementioned cognitive and structural barriers.

In summary, the interconnected structural and cognitive factors discussed above create a distinct learning context for art majors where engaging in English study involves substantial perceived challenges. These challenges stem from time conflicts with professional training and the high cognitive effort needed to cope with instructional mismatches. This distinctive context likely shapes the pathways through which motivational factors like task value relate to learning behaviors and outcomes in their EFL learning.

### The relationship between task value of learning English and English achievement

The mechanism through which task value influences academic achievement involves a nuanced interplay of direct and indirect pathways. The EVCT provides a strong foundational premise, positing that task value often operates primarily indirectly by shaping learning behaviors such as engagement and persistence [6,8]. Substantial evidence supports this view, showing that task value predicts academic outcomes both through the enhancement of learning strategies, effort, and self-efficacy, and by the reduction of learning procrastination [6,32–34].

However, empirical research has also identified conditions under which task value can directly predict academic achievement, suggesting that a strong, salient value perception may sometimes translate into outcomes without full mediation by proximal behaviors [35,36].

Integrating this evidence into the unique learning context of art majors sharpens the inquiry. While notable cognitive and structural barriers may dampen the translation of motivation into behavior [18,20,21], the perceived future utility of English could significantly drive art majors to have a strong overall task value for the language [20,21,30,31]. This raises the possibility that, alongside any indirect pathways, a strong and salient task value could maintain a direct motivational link to achievement even within a challenging environment [35–37]. Consequently, we first hypothesized a direct predictive effect:

H1. Art students’ task value of learning English positively predicts English achievement.

### The mediating role of learning engagement

Learning engagement represents a positive, fulfilling state of mind characterized by vigor, dedication, and absorption in academic tasks, reflecting a learner’s active and persistent involvement in the learning process [10,11,38–40]. The conceptualization and examination of engagement have extended beyond traditional classroom settings, with recent studies exploring its manifestations, antecedents, and dimensions in emerging learning contexts such as informal digital and AI-mediated environments [41–43]. Within EVCT, task value serves as a critical antecedent that fosters such engagement by enhancing students’ willingness to invest effort and persist in learning activities [3,6,16]. This motivational pathway is well-established in diverse educational settings. For instance, Vo and Ho [44] demonstrated in an online university setting that students’ task value beliefs significantly and positively predicted their learning engagement, a finding corroborated by longitudinal evidence from Vo et al. [10]. Similarly, in the context of hybrid MOOCs, Zhang et al. [11] found that task value belief directly promoted learner engagement.

Moreover, learning engagement itself functions as a robust predictor of academic achievement. Empirical studies across diverse EFL contexts have consistently linked higher engagement to better language learning outcomes [5,14,45]. For example, Luan et al. [14] and Sun and Zhang [5] both found engagement to be a significant predictor of English achievement among Chinese university students, while Jiang and Peng [45] highlighted the role of cognitive engagement in an LMOOC setting.

Taken together, these findings illustrate a widely supported general mechanism: task value enhances learners’ engagement, which in turn facilitates academic success. However, this established pathway has primarily been examined among non-art majors. The distinct learning context of art students, characterized by the structural and cognitive challenges outlined earlier [18,20,21], presents a critical boundary condition. It remains an open question whether and how this general mechanism functions when learners navigate such significant obstacles. Investigating the mediating role of learning engagement within this distinct population therefore constitutes a valuable extension of existing theory. Therefore, the following hypothesis was proposed:

H2. Learning engagement mediates the positive relationship between task value and English achievement among art majors.

### The mediating role of learning procrastination

Academic procrastination, the voluntary but irrational delay of academic tasks, is a prevalent self-regulatory failure that significantly threatens academic success [25,46–48]. Strong evidence confirms its negative impact on performance [49–52]. For instance, a learning analytics study linked procrastination to lower grades across tens of thousands of assignments [49], a finding corroborated by research in diverse student populations [52]. The tendency to procrastinate is closely linked to how learners value a task [6,8]. Related empirical research suggested that students are less likely to delay tasks they consider important, interesting, or useful [40], a view supported by negative correlations between task value and procrastination [25,50].

When considering art majors, it is essential to account for both their unique learning context and their motivational resources. The structural and cognitive barriers they face may increase the difficulty of English learning [20,21,29]. However, this group also maintains a strong perception of task value in learning English, which stems from recognizing the utility of English for global artistic engagement and career advancement [20,22,30,31]. Empirical evidence suggested that this salient, future-oriented task value can function as a motivational counterweight to the tendency to procrastinate, thereby motivating students to overcome immediate challenges in pursuit of long-term goals and consequently reducing delays [6,37,53]. Based on this reasoning, the following hypothesis was proposed:

H3. Learning procrastination mediates the relationship between task value and English achievement among art majors.

### The chain mediating role of learning engagement and learning procrastination

A more comprehensive mechanism linking task value to achievement can be elucidated by considering the sequential roles of learning engagement and procrastination. Empirical studies across diverse educational settings consistently demonstrated a robust negative association between learning engagement and academic procrastination [15,16]. For instance, Wang and Wang [15] identified learning engagement as a key mediator that translated perceived support into reduced procrastination among postgraduate students.

Grounded in EVCT, this negative relationship is logically preceded by task value, which initiates the entire behavioral sequence. As a well-established antecedent of learning engagement [6,44], task value motivates students to invest effort and immerse themselves in the learning process. This heightened engagement, in turn, serves as an effective alternative to procrastination, competing for the student’s time and cognitive resources, thereby reducing the likelihood of delay [54]. Recent evidence from technology-enhanced learning contexts further supported this chain, showing that interventions which boosted task value and engagement subsequently led to a significant reduction in academic procrastination [17].

When considering this chain mechanism for art majors, its operation presents a theoretically meaningful case. Their distinct learning environment, which involves salient time conflicts and cognitive demands, could potentially strain the link between engagement and reduced procrastination. Research on future-oriented motivation indicated that task value, when significantly driven by perceived professional utility, can establish a strong motivational foundation [6,37,53]. This logic may extend to art majors in the context of English learning. Thus, for this population, task value is expected not only to foster engagement but also to help sustain it, thereby enabling engagement to effectively function as a protective alternative to procrastination even within a challenging context. Therefore, based on this reasoning, these established links are hypothesized to outline a viable sequential mediation pathway for art majors: task value fosters heightened learning engagement, which subsequently functions as an active counterforce to learning procrastination, ultimately contributing to improved academic performance.

Integrating these insights, the following hypothesis was proposed:

H4. Learning engagement and learning procrastination sequentially mediate the relationship between task value and English achievement among art majors.

### The moderating role of self-efficacy

Academic self-efficacy, an individual’s conviction in their capability to organize and execute actions to achieve goals [55], plays a critical regulatory role in learning processes [2,18,34,55]. It functions as a key protective factor that buffers against the emergence of procrastination [13,56]. This regulatory function is particularly relevant to the pathway from engagement to procrastination. Students with high self-efficacy, fortified by confidence in their capabilities, are likely more effective at translating their learning engagement into persistent and focused effort. They are less prone to being derailed by challenges, thereby sustaining the negative impact of engagement on procrastination [13,33]. Conversely, for students with low self-efficacy, even periods of high engagement may be fragile; self-doubt and anxiety can undermine the quality and persistence of their effort, making them more susceptible to disengagement and delay when facing difficulties [2,35,57].

This established self-regulatory mechanism warrants specific examination within the distinct learning context of art majors. The unique challenges they face, such as managing high cognitive load due to instructional misalignment and balancing intense specialized training with academic requirements [18,20,29], create an environment where the ability to sustain engagement is constantly tested. It is plausible that self-efficacy plays a pivotal moderating role in this context [2,18,34].

Students with high self-efficacy are likely better equipped to leverage their engagement as a stable resource against procrastination, effectively navigating daily pressures and setbacks. Their confidence could be crucial for maintaining behavioral consistency when engagement is challenged by these difficulties. Conversely, for those with low self-efficacy, the same demanding environment might exacerbate the fragility of their engagement, more readily weakening its protective effect against procrastination. Thus, even within their unique learning environment, the regulatory function of self-efficacy on the engagement-procrastination link is expected to persist for art majors.

Therefore, the final hypothesis was proposed:

H5. Self-efficacy moderates the negative relationship between learning engagement and procrastination among art majors.

In summary, the reviewed literature firmly established the individual significance of task value, learning engagement, procrastination, and self-efficacy for academic outcomes, with EVCT offering a coherent explanatory framework. However, a critical synthesis of this work, particularly through the lens of art majors’ unique learning context, reveals important limitations in the current research landscape.

A primary concern pertains to external validity. Although empirical models detailing the interrelationships among these constructs have been predominantly developed and validated with non-arts majors [2,9,17], the constructs themselves are acknowledged as context-sensitive [3,10,22–25]. This sampling focus raises fundamental questions about the applicability of these models to a population characterized by structural and cognitive challenges [18,20,26]. Furthermore, and more critically, a significant limitation of prior research is its tendency to examine these variables in isolation or through simple pairwise relationships. A conspicuous absence remains of an integrated model capable of simultaneously investigating: (a) the sequential behavioral pathway (i.e., a chain mediation) via which task value translates into achievement, and (b) how this sequential process might be contingent upon a pivotal self-regulatory factor (i.e., moderated by self-efficacy). This state of research constrains a deeper understanding of the dynamic and conditional mechanisms at play, especially within a complex and distinct learning environment such as that of art students.

**Fig 1:**
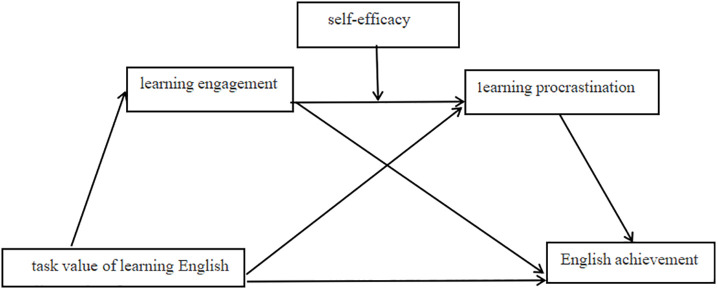
The hypothesized moderated chain mediation model.

To address these limitations, the present study proposed and tested a comprehensive moderated chain mediation model (see Fig 1). This model specified art students’ task value in learning English as the key antecedent. It posited learning engagement and learning procrastination as sequential mediators in the relationship between task value and English achievement. Furthermore, the model investigated self-efficacy as a moderator of the link between learning engagement and procrastination. The hypothesized relationships are illustrated in Fig 1: H1 proposed that art students’ task value of learning English positively predicts English achievement; H2 posited that learning engagement mediates the positive relationship between task value and English achievement among art majors; H3 proposed that learning procrastination mediates the relationship between task value and English achievement among art majors; H4 hypothesized that learning engagement and learning procrastination sequentially mediate the relationship between task value and English achievement among art majors; and H5 proposed that self-efficacy moderates the negative relationship between learning engagement and procrastination among art majors. By examining these specific, integrated pathways, the study aimed to elucidate the contextualized motivational dynamics among art majors and to test the explanatory power of EVCT within a distinct learning environment.

## Method

### Participants

Participants were recruited through convenience sampling from 12 classes at a university in China. The inclusion criteria were: (a) officially enrolled as art majors; (b) in their second year of study and had completed at least one year of foundational college English instruction as part of their general education requirements; and (c) voluntarily agreed to participate after being fully informed of the study’s purpose and procedures. The initial data collection yielded 633 responses. After excluding 45 responses due to incomplete questionnaires or patterned responses (e.g., straight-lining), the final analytical sample consisted of 588 participants (445 females, 75.7%; 143 males, 24.3%), with ages ranging from 18 to 29 years (*M* = 19.58, *SD* = 0.83).

Regarding their English learning experience, the majority of participants (71.1%) reported starting English learning in the third grade of primary school, followed by those who started in first grade (25.2%) and first year of junior high school (3.7%). The mean years of English learning for each discipline were as follows: painting (n = 101, *M* = 11.31, *SD* = 1.95), drama and film studies (n = 74, *M* = 11.81, *SD* = 2.08), calligraphy (n = 91, *M* = 11.72, *SD* = 1.86), dance (n = 129, *M* = 11.61, *SD* = 2.20), visual communication design (n = 114, *M* = 11.77, *SD* = 2.17), and aviation service arts and management (n = 79, *M* = 11.76, *SD* = 1.99). The distribution of English learning experience was comparable across disciplines, indicating that the sample was appropriate for examining the proposed relationships without confounding effects of disciplinary differences.

### Instruments

This study used an online questionnaire to collect data. The questionnaire consisted of two sections: the first collected demographic information, including gender, age, major, grade, English learning experiences, and cumulative time spent learning English; the second comprised four scales measuring task value, learning engagement, learning procrastination, and self-efficacy. With the exception of the learning procrastination scale, all scales used in this study were originally developed as 7-point Likert-type instruments. To maintain consistency across all measures and reduce respondent fatigue, they were adapted to a uniform 5-point Likert scale ranging from 1 (strongly disagree) to 5 (strongly agree). The item wording remained unchanged from the original versions to preserve content validity.

To ensure conceptual and linguistic appropriateness for Chinese participants, the questionnaire was administered in Chinese. All instruments underwent a rigorous translation and back-translation process. First, two bilingual research assistants independently translated the scales into Chinese and then back-translated them into English. Subsequently, a panel of four experts in psychology and foreign languages reviewed the translated versions, compared them with the originals, resolved discrepancies, and ensured content validity. The final versions of all scales demonstrated good to excellent reliability and construct validity, as detailed in the following subsections.

### Measurement of task value of learning English

Task Value Scale (TV-S), comprising 9 items, was selected from the Motivated Strategies for Learning Questionnaire (MSLQ) [55]. It was used to measure students’ task value in learning English (e.g., assessing interest in the content, perceived importance, and preference for challenge). The Chinese version of the TV-S demonstrated excellent internal consistency, with a Cronbach’s alpha of.903. Confirmatory factor analysis (CFA) supported the construct validity of the scale: χ²/df = 2.753, CFI = .981, NFI = .971, IFI = .981, RMSEA = .055. The standardized factor loadings for all items ranged from.631 to.799.

### Measurement of learning Engagement

Learning Engagement Scale (LE-S) was taken from the Utrecht Work Engagement Scale-Student (UWES-S) developed by Schaufeli et al. [38]. It comprises 17 items grouped into three dimensions: vigor (e.g., “When I get up in the morning, I feel like going to class”), dedication (e.g., “I am enthusiastic about my studies”), and absorption (e.g., “When I am studying, I forget everything else around me”). The Chinese version of the scale demonstrated good internal consistency, with a Cronbach’s alpha of.932. CFA showed that the scale had good construct validity, with the following indices: χ²/df = 4.076, CFI = .923, NFI = .901, IFI = .923, and RMSEA = .072. The standardized factor loadings for all items ranged from.609 to.731.

### Measurement of learning procrastination

The Learning Procrastination Scale (LP-S), developed by McCloskey and Scielzo [59], was employed to assess students’ procrastination behaviors. This 25-item instrument covers six dimensions: psychological beliefs related to procrastination (e.g., “I know I should work on school work, but I just don’t do it”), distraction (e.g., “I get distracted by other, more fun things when I am supposed to work on schoolwork”), social factors (e.g., “On the weekends, I make plans to do homework and projects, but I get distracted and hang out with friends”), time management (e.g., “I allocate time so I don’t have to cram at the end of the semester”), personal initiative (e.g., “I tend to put off things for the next day”), and laziness (e.g., “I don’t spend much time studying school material until the end of the semester”). Items 1, 8, 12, 14, and 25 are reverse-scored. The Chinese version of the LP-S demonstrated excellent internal consistency, with a Cronbach’s alpha of.950. CFA indicated acceptable model fit for construct validity, with the following indices: χ²/df = 3.680, CFI = .901, TLI = .892, IFI = .901, and RMSEA = .068. The standardized factor loadings for all items ranged from.609 to.736.

### Measurement of self-efficacy

Self-Efficacy Scale (SE-S), comprising 9 items, was adopted from the Motivated Strategies for Learning Questionnaire (MSLQ) developed by Pintrich and De Groot [58]. It was used to assess students’ perceived capability and confidence in performing class assignments (e.g., “Compared with other students in this class I expect to do well”). The Chinese version of the scale used in this study demonstrated high internal consistency, with a Cronbach’s alpha of.892. CFA indicated acceptable model fit for construct validity, with the following indices: χ²/df = 3.44, CFI = .971, NFI = .960, IFI = .971, and RMSEA = .065. The standardized factor loadings for all items ranged from.639 to.741.

### Measurement of English achievement

Influenced by academic achievement-prioritized environments, English test scores are often regarded by Chinese EFL learners as an important indicator of their language proficiency [2,14,60].Therefore, the end-of-term examination was used as an indicator of art students’ applied English language ability. The term-final exam was designed with reference to the College English Test Band 4, a nationwide standardized English exam in China, and was jointly developed by an external expert panel in English language teaching. The difficulty of the test paper was comprehensively evaluated by a team of art English instructors from the participants’ university before its administration, ensuring adequate reliability and validity of the instrument used in this study. The test paper consisted of six sections: multiple-choice listening exercises, fill-in-the-blank reading exercises, multiple-choice reading exercises, paragraph matching, paragraph translation, and essay writing. The objective items in the first four sections were scored by computer, while the translation and writing parts were graded collectively through a collaborative and division-based marking process by the university’s English instructors to ensure scoring rigor. Participants were required to complete the test within 120 minutes, and the maximum score was 100 points.

### Data collection

This study received ethical approval from the Biomedical Ethics Committee of Qufu Normal University (Approval No.: 2025098) and was conducted in accordance with the Declaration of Helsinki. Prior to data collection, all participants were fully informed about the study’s purpose, data anonymity, and their right to withdraw without penalty. Electronic informed consent was mandatory and had to be provided via the online survey platform before accessing the questionnaire. For participants under 18, parental/guardian permission was secured in addition to the participant’s own assent. All data were collected and analyzed anonymously.

The research was conducted in two phases. A pilot study was first launched on December 9, 2024, with 223 invited participants. After data cleaning, 200 valid responses were retained to refine the survey instruments; these data were not included in the formal analysis. The formal study then commenced on December 16, 2024, using the revised questionnaire distributed via “wjx.cn” until December 30, 2024. From the 633 participants targeted in this phase, 588 valid questionnaires were retained after the removal of incomplete and straight-line responses, resulting in a valid response rate of 92.89%.

### Data analysis

Data analysis followed a three-step procedure using SPSS 26.0, AMOS 24.0, and PROCESS. First, the reliability and construct validity of the scales were examined. Then, common method bias testing, descriptive statistics, and correlation analysis were conducted. Finally, path analysis was employed to examine the relationships among constructs. Specifically, the chain mediation effect was tested using PROCESS macro for SPSS (Model 6) with the bias-corrected percentile bootstrap method (5,000 resamples, 95% CI) [61]. To evaluate the moderated chain mediation, a moderating variable was introduced without altering previous variables. After mean-centering the data, PROCESS Model 91 was applied to assess this model [62,63].

## Results

### Common method deviation test

As the results may be influenced by common method bias due to the use of self-reported questionnaires, the Harman single-factor test was employed to assess common method bias [64]. An unrotated factor analysis of all survey items yielded 6 factors with eigenvalues greater than one. The first factor accounted for 32.775% of the variance, which was below the critical threshold of 40% [64]. These results suggested that no severe common method bias was present in the data.

### Descriptive statistics and correlation analysis

Table 1 presented the descriptive statistics and correlation coefficients for all study variables. The analysis revealed significant positive correlations among task value of learning English, learning engagement, self-efficacy, and English achievement, while learning procrastination was significantly negatively correlated with all of these variables. The significant correlations among the variables satisfied the prerequisite for testing mediation effects. Additionally, gender was positively correlated with self-efficacy. Major was significantly positively correlated with task value of learning English. Therefore, gender, major were included as control variables in subsequent analyses.

**Table 1:** Descriptive statistics and correlations among main variables.

Variable	*M*	*SD*	1	2	3	4	5	6	7	8
1 Gender			1							
2 Major			−.076	1						
3 TTSLE			−.027	.056	1					
4 EA	65.090	11.007	.010	−.027	.010	1				
5 TVLE	3.819	0.665	.075	.102^*^	.029	.155^**^	1			
6 LE	2.842	.648	.055	.068	−.005	.378^**^	.289^**^	1		
7 SE	2.892	.733	.103^*^	−.031	−.050	.410^**^	.298^**^	.696^**^	1	
8 LP	3.289	.613	−.071	.014	.056	−.481^**^	−.208^**^	−.636^**^	−.618^**^	1

Note. N = 588; ^*^p < 0.05, ^**^p < 0.01; TTSLE: Total time spent learning English; EA: English achievement; TVLE: task value of learning English; LE: learning engagement; SE: self-efficacy; LP: learning procrastination

### Chain mediation effect analysis

The results (Table 2) showed that task value of learning English significantly and positively predicted learning engagement (*β* = .282, *p* < .001). However, task value of learning English did not significantly predict learning procrastination (*β* = –.029, *p* > .05). Learning engagement significantly and negatively predicted learning procrastination (*β* = –.630, *p* < .001). When task value of learning English, learning engagement, and learning procrastination were included together in the model predicting English achievement, learning engagement significantly and positively predicted English achievement (*β* = .115, *p* < .05), while learning procrastination significantly and negatively predicted English achievement (*β* = –.400, *p* < .001). At this stage, task value of learning English was no longer a significant direct predictor of English achievement (*β* = .405, *p* > .05).

**Table 2:** Test for the chain mediation model.

Regression equation		Overall fit indices			Significance of regression Coefficients (std.)		
Result variables	Predictive variables	*R*	*R* ^*2*^	*F*	*β*	*95%C*	*t*
LE	TVLE	.294	.087	18.436***	.282	[.199,.351]	7.075***
LP	TVLE	.640	.401	101.082***	−.029	[-.087,.034]	−.865
	LE				−.630	[-.658, -.534]	−18.917***
EA	TVLE	.493	.243	37.465***	.405	[-.488, 1.978]	1.187
	LE				.115	[.359, 3.557]	2.406*
	LP				−.400	[-8.828, -5.521]	−8.521***

Note. N=588; *p < 0.05, **p < 0.01, ***p < 0.001

The mediation effect analysis (Table 3) showed that the indirect effect via learning engagement was.033, that via learning procrastination was.012, and the chain mediation effect via both learning engagement and learning procrastination was.071. The bootstrap 95% confidence intervals for the mediation path through learning engagement and the chain mediation path through learning engagement and learning procrastination did not include zero, indicating that these indirect effects were statistically significant. In contrast, the mediating effect through learning procrastination was not significant, as its 95% confidence interval included zero.

**Table 3:** Mediating effect analysis of the chain mediating model.

	*Effect*	*SE*	*95% CI*
Total effect	.160	.682	[1.313, 3.992]
Direct effect	.045	.628	[-.488, 1.978]
Total indirect effect	.115	.021	[.073,.156]
Mediating effect of LE	.033	.015	[.005,.063]
Mediating effect of LP	.012	.014	[-.015,.039]
Chain mediating effect of LE and LP	.071	.014	[.044,.099]

Note. N = 588;SE and 95% CI refer to the standard errors, lower and upper 95% confidence intervals of the indirect effects estimated by the bias-corrected percentile Bootstrap method, respectively

These results provide support for H2 (learning engagement mediates the relationship between task value and English achievement) and H4 (learning engagement and learning procrastination sequentially mediate this relationship), while H1 (task value directly predicts English achievement) and H3 (learning procrastination independently mediates this relationship) were not supported.

### Moderated chain mediation effect analysis

The results (Table 4) indicated that, after controlling for relevant variables, the path coefficients of the model showed mixed significance. Task value of learning English had a significant positive predictive effect on learning engagement (*β* = .282, *p* < 0.001). Learning engagement demonstrated a significant negative predictive effect on learning procrastination (*β* = –.373, *p* < 0.001). The interaction term between learning engagement and self-efficacy also had a significant negative predictive effect on learning procrastination (*β* = –.087, *p* < 0.001). Furthermore, learning engagement showed a significant positive predictive effect on English achievement (*β* = .115, *p* < 0.05), while learning procrastination had a significant negative predictive effect on English achievement (*β* = –.400, *p* < 0.001). On the other hand, task value of learning English did not significantly predict learning procrastination (*β* = .011, *p* > 0.05) or English achievement (*β* = .045, *p* > 0.05).

**Table 4:** Test for the moderated chain mediation model.

Regression equation		Overall fit indices			Significance of regression Coefficients (std.)		
Outcome variables	Predictors	*R*	*R* ^*2*^	*F*	*β*	*95% CI*	*t*
LE	TVLE	.294	.087	18.436***	.282	[.204,.360]	7.075***
LP	TVLE	.689	.475	87.617***	.011	[-.052,.073]	.325
	LE				−.373	[-.458, -.287]	−8.549***
	LE × SE				−.087	[-.138, -.036]	−3.323***
EA	TVLE	.493	.243	37.465***	.045	[-.030,.120]	1.187
	LE				.115	[.021,.209]	2.406*
	LP				−.400	[-.492, -.308]	−8.521***

Note: N = 588.*p < 0.05, **p < 0.01,***p < 0.001

**Fig 2:**
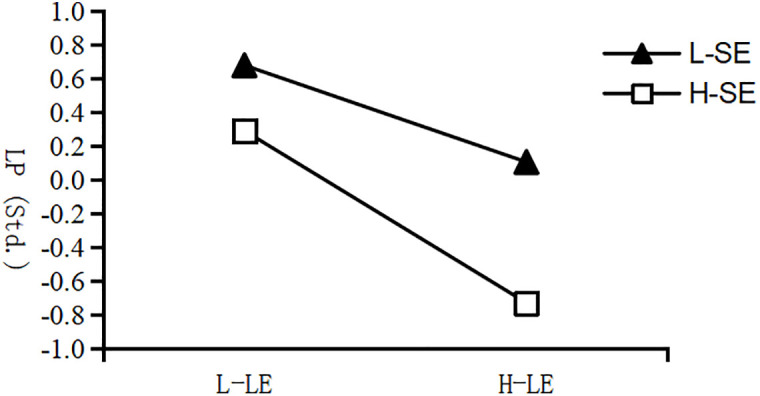
The moderating effect of self-efficacy on learning engagement and procrastination. L-SE: low self-efficacy; H-SE: high self-efficacy; L-LE: low learning engagement; H-LE: high learning engagement; LP: learning procrastination.

To further probe the interaction, a simple slope analysis was performed. As depicted in Fig 2, increased learning engagement was linked to lower learning procrastination at both levels of self-efficacy. However, this negative relationship was significantly stronger for individuals with high self-efficacy, demonstrated by a steeper slope, compared to those with low self-efficacy. This pattern confirmed that self-efficacy acted as a significant moderator, strengthening the negative impact of learning engagement on learning procrastination.

These results provide support for H5 (self-efficacy moderates the negative relationship between learning engagement and procrastination), while further confirming the support for H2 and H4 and the lack of support for H1 and H3 as reported in the chain mediation analysis.

## Discussion

Focusing on art majors in Chinese universities, this study employed the EVCT to investigate the role of task value in their unique learning context. By testing a moderated chain mediation model, it systematically analyzed the relationships among task value of learning English, learning engagement, learning procrastination, self-efficacy, and English achievement. The findings partially supported the hypothesized model, clarifying the motivational and behavioral dynamics in this population’s English learning process.

### The relationship between task value of learning English and English achievement

Contrary to H1, task value of learning English did not demonstrate a significant direct association with English achievement among art majors. This finding, however, helps clarify the specific nature of the relationship between these variables within this unique population. The results are consistent with the core proposition of EVCT, which posits task value as a key antecedent of learning behaviors [6,8]. Furthermore, they highlight how the learning context can shape the pathways through which motivation is associated with academic outcomes.

This pattern of results can be understood by integrating our findings with the distinct learning context of art majors established earlier. The literature suggests that art students’ learning is situated within an environment characterized by a positive appraisal of English learning tasks, particularly their perceived utility for professional development [6,20,37,53], alongside significant structural and cognitive challenges. These include difficulties arising from competing academic priorities, limited time allocated to English learning [20,21], and a cognitive mismatch between their visual-practical learning preferences [18,27] and the verbal-logical demands of traditional English instruction [28]. Our empirical model indicated that within this specific context, the relationship between task value and achievement was fully accounted for by its sequential linkages with learning engagement and procrastination. Specifically, task value was positively related to engagement, a connection supported by prior research [6,44]. Engagement, in turn, showed a strong negative association with procrastination [15,16], which itself was significantly negatively correlated with achievement [49,52].

In summary, the path analysis supports a model in which the association between task value and English achievement is fully conveyed through this behavioral sequence, rather than being direct. This pattern suggests that for art majors, the role of task value is more proximal to the initiation and maintenance of learning behaviors than to the direct association with learning outcomes. Therefore, the non-significant direct relationship does not diminish the importance of task value but rather specifies its function: it appears to be a critical precursor that is linked to outcomes through a chain of intermediary learning processes [6,32].

### The mediating role of learning engagement

The significant mediating role of learning engagement revealed a key behavioral mechanism linking art majors’ task value to academic achievement. Although art majors report lower intrinsic interest in English courses [20,21], the established mediation pathway suggests that their overall task value is effectively channeled through learning engagement. This task value is potentially driven by a recognition of its pervasive importance for academic and professional advancement [30,31,37,53].

Drawing on existing literature, it is plausible that this future-oriented task value, which is associated with career development [6,20,37,53], is linked to a strong and sustained behavioral drive. This drive is characterized by the vigor, dedication, and absorption of learning engagement. In turn, engagement is associated with the capacity to overcome the combined challenges of structural constraints, motivational barriers, and cognitive mismatches that characterize their learning environment [19,27,28].

In essence, for art majors, task value is linked to higher achievement primarily through initiating and sustaining deep cognitive and behavioral immersion. Thus, engagement emerges as a pivotal mediating process. This process reflects a pathway through which a primarily career-driven valuation of English learning tasks may be associated with academic success [6,20,37,53].

### The mediating role of learning procrastination

H3 was not supported, as learning procrastination did not serve as a significant independent mediator between task value and English achievement. This finding indicates that while task value is positively associated with learning engagement, a direct link from task value to reduced procrastination is not observed.

The absence of an independent mediating role for procrastination can be interpreted within the study’s framework. This study measured task value as a positive appraisal, situated within a learning environment characterized by notable challenges. In this specific context, the motivational force of task value alone appears insufficient to directly counteract a strong tendency to procrastinate. The substantial structural and cognitive barriers faced by art majors [18,20,29] may attenuate any direct inhibitory effect positive task value might have on procrastination. Consequently, task value of learning English did not show a robust direct association with procrastination.

Therefore, within this challenging context, task value of learning English does not shortcut to directly reduce delay. Instead, the observed negative relationship between task value of learning English and procrastination was fully channeled through the pathway of learning engagement. This suggests that students who became more immersed in learning tended to exhibit less procrastinatory behavior [15,16].

This pattern helps explain why procrastination, while negatively correlated with achievement [49,52], does not function as an independent mediator. It underscores that for art majors, task value may need to first be translated into substantial behavioral engagement to generate sufficient motivational momentum to effectively curb procrastination within a demanding environment.

### The chain mediating role of learning engagement and learning procrastination

This study supported H4, indicating a significant chain mediation pathway in which task value of learning English was positively associated with learning engagement, which in turn was related to lower levels of procrastination, and ultimately to English achievement. This sequential pattern is consistent with prior research indicating that value beliefs are linked to achievement through behavioral channels [6,8].

The identified chain mediation may be understood within art students’ specific distinct learning context. While art majors face challenges including limited instructional time [20,21] and cognitive mismatches [18,27,28], task value of learning English is linked to the initiation of a productive behavioral sequence. The initial link between task value of learning English and learning engagement suggests that students’ valuation of English learning, potentially driven by its perceived future relevance [6,20,37,53], is associated with a willingness to invest effort despite obstacles [6,44]. Subsequently, the linkage from learning engagement to reduced procrastination reflects how active involvement is associated with a decrease in avoidance tendencies, a pattern documented across diverse contexts [15,16]. For art majors specifically, the immersive quality of learning engagement may mitigate the difficulty of English learning by providing immediate task involvement, thereby reducing opportunities for delay.

The complete mediation through this behavioral sequence suggests that for art majors, task value of learning English functions primarily as an instigator of productive learning processes rather than being directly linked to outcomes. This indirect pathway illustrates the crucial role of translating motivational resources into sustained behavioral involvement, particularly in learning environments characterized by structural and cognitive constraints.

### The moderating role of self-efficacy

This study confirmed H5, indicating that self-efficacy significantly moderated the negative relationship between learning engagement and procrastination. This finding extends the understanding of self-efficacy as a key regulatory mechanism to the distinct learning context of art majors [13,56].

This moderating effect can be interpreted by considering how art students with different self-efficacy levels navigate their specific challenges. Given their intensive specialized training schedules [20], art majors must balance professional practice with academic demands. Under these structural constraints, students with higher self-efficacy appear more capable of strategically protecting their English learning time. This may enable them to maintain the consistent effort prompted by engagement, which is associated with lower levels of procrastination [2]. In contrast, those with lower self-efficacy may struggle more to navigate these competing demands, potentially leading to overwhelmed feelings and greater difficulty in sustaining consistent learning behaviors despite initial engagement.

Similarly, when confronting the cognitive mismatch inherent in their learning environment [18,27,28], students with high self-efficacy are more likely to perceive these difficulties as manageable challenges, exhibiting greater behavioral persistence [33]. Conversely, students with low self-efficacy may be more prone to perceive the same obstacles as threatening, a perception linked to heightened anxiety and a greater likelihood of early disengagement [57]. This divergence helps explain why the regulatory role of self-efficacy is particularly salient for art majors.

The stronger negative relationship observed among high self-efficacy students highlights how confidence beliefs can serve as crucial psychological resources within a demanding learning environment. For art majors, self-efficacy may act as a critical buffer, associated with sustaining engaged behaviors despite structural pressures and cognitive obstacles. This protective function underscores the importance of fostering self-efficacy alongside task value in supporting art students’ English learning.

### Theoretical and pedagogical implications

This study contributes to the EVCT framework and the broader literature on L2 motivation in several ways. By investigating the motivational dynamics of art majors, a population facing unique structural and cognitive challenges in EFL learning, this study extends the application of EVCT to an underexplored context. The findings demonstrate that the core propositions of EVCT hold within this distinct population, thereby addressing concerns regarding the external validity of existing motivational models that have been predominantly developed and validated with non-arts majors. The observed pathways through which task value relates to English achievement via sequential mediation of learning engagement and procrastination suggest that the framework’s explanatory power generalizes beyond traditionally studied populations.

The study also advances an integrated moderated chain mediation model that moves beyond the isolated or pairwise examinations that have characterized prior research. By simultaneously examining the sequential mediating roles of learning engagement and procrastination, as well as the moderating role of self-efficacy, the findings provide a more nuanced understanding of how motivational processes unfold in context. The identification of self-efficacy as a boundary condition of the engagement procrastination relationship contributes to theoretical refinement by specifying when and for whom engagement is most effective in reducing procrastination. This conditional effect highlights the importance of considering individual differences in self-regulatory resources within the EVCT framework.

Furthermore, the study empirically validates the chain mediation mechanism linking task value to achievement through engagement and procrastination, offering empirical support for the theoretical assumption that task value operates primarily through behavioral pathways rather than directly influencing outcomes. This finding aligns with and extends EVCT’s core proposition by demonstrating the sequential nature of these pathways in a challenging learning environment.

These theoretical contributions have important pedagogical implications for EFL instruction tailored to art majors. To enhance task value, instructors could create direct connections between language learning and students’ artistic domains and future careers [6,20,37,53], for instance, by designing tasks such as researching international art movements or preparing English descriptions for creative portfolios. To support the link between learning engagement and reduced procrastination, learning environments could promote active involvement through structured routines, collaborative activities, and timely feedback [15]. Given art students’ cognitive preferences for visual and experiential learning [18,27], teaching methods can be adapted by incorporating multimedia, project-based learning, or role-playing, which may help reduce extraneous cognitive load and better align with students’ strengths [29]. Additionally, fostering self-efficacy is important to support sustained engagement. Providing structured support, such as writing guides and speaking templates, along with positive feedback could assist students, particularly those with lower confidence, in managing tasks more effectively [33,57].

In summary, a comprehensive instructional approach that aims to cultivate task value, promote active engagement through contextually aligned methods, and build self-efficacy may help create conditions associated with improved learning outcomes for art majors.

## Conclusion

This research examined the relationship between task value of learning English and English achievement among art majors by testing a moderated chain mediation model. The findings revealed that task value was associated with English achievement entirely through indirect pathways. Specifically, task value was linked to higher learning engagement, which in turn was associated with lower levels of procrastination, with both engagement and procrastination further linked to English achievement. Furthermore, self-efficacy moderated the negative relationship between learning engagement and procrastination, with this association being stronger among students with higher self-efficacy. These findings illustrate the interconnected roles of motivational, behavioral, and self-regulatory factors in the English learning of art students within their unique learning context.

Despite these contributions, several limitations should be acknowledged. The cross-sectional nature of the data precludes causal inferences, and the sample was drawn exclusively from art majors at a single university in China, which may limit generalizability. Task value was measured as a unidimensional construct, which does not capture the potential distinct roles of its subdimensions, and information regarding specific instructional methods experienced by participants was not collected.

Building on these limitations, future research should employ longitudinal designs to trace the developmental trajectories of these constructs over time. Replication studies across different institutional and regional contexts would help establish the boundary conditions of the proposed model. A multidimensional approach to measuring task value could yield deeper insights into the unique contributions of attainment, intrinsic, and utility value. Finally, incorporating contextual data on instructional approaches, perhaps through classroom observations or interviews, would enrich understanding of how pedagogical factors interact with the motivational dynamics identified in this study.

## Supporting information

S1 File Survey instruments used in the study. The complete set of survey items, measuring task value of learning English, learning engagement, learning procrastination, and self-efficacy.
(DOCX)

S2 Data Anonymous dataset underlying the findings. The processed and anonymized data file containing all variables analyzed in this study.
(XLSX)
