# Supplementary material for: The relationship between task value of learning English and English achievement among art majors: A moderated chain mediation model analysis
Source: PLoS One. 2026 May 15;21(5):e0349225. doi: 10.1371/journal.pone.0349225 (PMC13178886; doi:10.1371/journal.pone.0349225)
Supplement: S1 File — The complete set of survey items, measuring task value of learning English, learning engagement, learning procrastination, and self-efficacy. (DOCX) [file pone.0349225.s001.docx]

**Items for task value of learning English (**TV-S**)**

TV-S1: It is important for me to learn what is being taught in this class.

TV-S2: I like what I am learning in this class.

TV-S3: I prefer class work that is challenging so I can learn new things.

TV-S4: I think I will be able to use what I learn in this class in other classes.

TV-S5: I often choose paper topics that allow me to learn something even though they require a lot

of time.

TV-S6: Even when I do poorly on a test I try to learn from my mistakes.

TV-S7: I think that what I am learning in this class is useful for me to know.

TV-S8: I think that what I am learning in this class is interesting.

TV-S9: Understanding this subject is important to me.

**Items for learning engagement (LE-S)**

LE-S1: When I get up in the morning, I feel like going to class.

LE-S2: When I’m doing my work as a student, I feel bursting with energy.

LE-S3: As far as my studies are concerned I always persevere, even when

things do not go well.

LE-S4: I can continue studying for very long periods at a time.

LE-S5: I am very resilient, mentally, as far as my studies are concerned.

LE-S6: I feel strong and vigorous when I’m studying or going to class.

LE-S7: To me, my studies are challenging.

LE-S8: My study inspires me.

LE-S9: I am enthusiastic about my studies.

LE-S10: I am proud of my studies.

LE-S11: I find my studies full of meaning and purpose.

LE-S12: When I am studying, I forget everything else around me.

LE-S13: Time flies when I am studying.

LE-S14: I get carried away when I am studying.

LE-S15: It is difficult to detach myself from my studies.

LE-S16: I am immersed in my studies.

LE-S17: I feel happy when I am studying intensely.

**Items for learning procrastination (LP-S)**

LP-S1: I usually allocate time to review and proof read my work.*

LP-S2:I put off projects until the last minute.

LP-S3:I have found myself waiting until the day before to start a big project.

LP-S4:I know I should work on school work, but I just don’t do it.

LP-S5:When working on schoolwork, I usually get distracted by other things.

LP-S6:I waste a lot of time on unimportant things.

LP-S7:I get distracted by other, more fun things when I am supposed to work on schoolwork.

LP-S8:I concentrate on school work instead of other distractions. *

LP-S9: I can’t focus on school work or projects for more than an hour until I get distracted.

LP-S10:My attention span for schoolwork is very short.

LP-S11:Tests are meant to be studied for just the night before.

LP-S12:I feel prepared well in advance for most tests. *

LP-S13: “Cramming” and last minute studying is the best way that I study for a big test.

LP-S14:I allocate time so I don’t have to cram at the end of the semester. *

LP-S15:I only study the night before exams.

LP-S16:If an assignment is due at midnight, I will work on it until 11:59.

LP-S17:When given an assignment, I usually put it away and forget about it until it is almost due.

LP-S18:Friends usually distract me from schoolwork.

LP-S19: I find myself talking to friends or family instead of working on school work.

LP-S20:On the weekends, I make plans to do homework and projects, but I get distracted and hang out with friends.

LP-S21: I tend to put off things for the next day.

LP-S22:I don’t spend much time studying school material until the end of the semester.

LP-S23: I frequently find myself putting important deadlines off.

LP-S24:If I don’t understand something, I’ll usually wait until the night before a test to figure it out.

LP-S25:I read the textbook and look over notes before coming to class and listening to a lecture or teacher. *

Note.items marked with * are reverse-scored.

**Items for self-efficacy (SE-S)**

SE-S1: Compared with other students in this class, I expect to do well.

SE-S2: I’m certain I can understand the ideas taught in this course.

SE-S3: I expect to do very well in this class.

SE-S4: Compared with others in this class, I think I’m a good student.

SE-S5: I am sure I can do an excellent job on the problems and tasks assigned for this class.

SE-S6: I think I will receive a good grade in this class.

SE-S7: My study skills are excellent compared with others in this class.

SE-S8: Compared with other students in this class, I think I know a great deal about the subject.

SE-S9: I know that I will be able to learn the material for this class.
